# The acceptability and feasibility of using the Adult Social Care Outcomes Toolkit (ASCOT) to inform practice in care homes

**DOI:** 10.1186/s12913-016-1763-1

**Published:** 2016-09-29

**Authors:** Ann-Marie Towers, Nick Smith, Sinead Palmer, Elizabeth Welch, Ann Netten

**Affiliations:** Personal Social Services Research Unit (PSSRU), University of Kent, Canterbury, CT2 7NF UK

**Keywords:** Outcomes, Quality of life, Social care, Care homes, Older people, Dementia, ASCOT

## Abstract

**Background:**

The Adult Social Care Outcomes Toolkit (ASCOT) measures social care related quality of life (SCRQoL) and can be used to measure outcomes and demonstrate impact across different social care settings. This exploratory study built on previous work by collecting new inter-rater reliability data on the mixed-methods version of the toolkit and exploring how it might be used to inform practice in four case study homes.

**Method:**

We worked with two care home providers to agree an in-depth study collecting SCRQoL data in four case-study homes. Data was collected about residents’ age, ethnicity, cognitive impairment, ability to perform activities of daily living and SCRQoL in the four homes. Feedback sessions with staff and managers were held in the homes two weeks after baseline and follow-up data collected three months later. Interviews with managers explored their views of the feedback and recorded any changes that had been made because of it.

**Results:**

Participant recruitment was challenging, despite working in partnership with the homes. Resident response rates ranged from 23 to 54 % with 58 residents from four care homes taking part in the research. 53 % lacked capacity to consent. Inter-rater reliability for the ASCOT ratings of SCRQoL were good at time one (IRR = 0.72) and excellent at time two (IRR = 0.76). During the study, residents’ ability to perform activities of daily living declined significantly (z = -2.67, *p* < .01), as did their expected needs in the absence of services (z = -2.41, *p* < .05). Despite these rapid declines in functionings, residents’ current SCRQoL declined slightly but not significantly (Z = -1.49, *p* = .14). Staff responded positively to the feedback given and managers reported implementing changes in practice because of it.

**Conclusion:**

This exploratory study faced many challenges in the recruitment of residents, many of whom were cognitively impaired. Nevertheless, without a mixed-methods approach many of the residents living in the care homes would have been excluded from the research altogether or had their views represented only by a representative or proxy. The value of the mixed-methods toolkit and its potential for use by providers is discussed.

**Electronic supplementary material:**

The online version of this article (doi:10.1186/s12913-016-1763-1) contains supplementary material, which is available to authorized users.

## Background

In many countries, central governments are responding to the challenges of an ageing population. With the proportion of people requiring long-term care expected to increase, policy makers are keen to deliver health and social care services efficiently and effectively. In the UK, the importance of measuring people’s outcomes, wellbeing and quality of life to support service evaluation and planning has been emphasised by researchers and accepted by policymakers and service providers for some time. Work in this area has developed considerably, particularly in terms of the development of measures for research and economic evaluation [1]. In England, national outcomes frameworks have been developed for adult social care (the Adult Social Care Outcomes Framework) [2] and the Care Act [3] has placed a statutory responsibility on local government to place well-being at the heart of care and support [4]. Care homes will increasingly be expected to demonstrate the impact and quality of the care and support they provide, as the regulator asks whether services are; safe, effective, caring, responsive and well-led [5]. The Adult Social Care Outcomes Toolkit (ASCOT) was derived through a series of studies and to date is the only measure focusing specifically on the areas of quality of life that can reasonably be attributed to social care services [6]. ASCOT is a preference-weighted measure with eight conceptually distinct domains of social care related quality of life (SCRQoL), outlined in Table 1. The domains cover the basic (personal cleanliness and comfort, accommodation cleanliness and comfort, food and drink, and feeling safe) and higher order (social participation, occupation, and control over daily life) aspects of SCRQoL. The final domain, dignity, differs from the other domains, reflecting the impact of the care process on how people feel about themselves [7].

**Table 1:** The ASCOT domains

Domain	Definition
Control over daily life	The service user can choose what to do and when to do it, having control over his/her daily life and activities
Personal cleanliness and comfort	The service user feels he/she is personally clean and comfortable and looks presentable or, at best, is dressed and groomed in a way that reflects his/her personal preferences
Food and drink	The service user feels he/she has a nutritious, varied and culturally appropriate diet with enough food and drink he/she enjoys at regular and timely intervals
Personal safety	The service user feels safe and secure. This means being free from fear of abuse, falling or other physical harm
Social participation and involvement	The service user is content with their social situation, where social situation is taken to mean the sustenance of meaningful relationships with friends, family and feeling involved or part of a community should this be important to the service user
Occupation	The service user is sufficiently occupied in a range of meaningful activities whether it be formal employment, unpaid work, caring for others or leisure activities
Accommodation cleanliness and comfort	The service user feels their home environment, including all the rooms, is clean and comfortable
Dignity	The negative and positive psychological impact of support and care on the service user’s personal sense of significance

The ASCOT domains were identified through expert review with professional stakeholders to ensure its sensitivity to outcomes of interest to policymakers and its relevance to the evaluation of social care interventions [6]. This was complemented by a literature review exploring service users’ understanding of social care outcomes and cognitive interviews to check social care service users’ understanding of terms and to clarify the wording of the items [6]. Although ASCOT is not a measure of health outcomes, previous research has found a reasonable relationship with the EQ-5D (*r* = 0.4) [7], whilst also confirming that ASCOT is more sensitive to the impact of social care interventions, such as care provided in people’s own homes to help them get up, washed, dressed, eat meals and keep their home clean and comfortable [7–9].

Although developed in the UK, since its release in 2012, ASCOT has received a lot of international attention and has been used by researchers in studies examining the impact of various forms of long-term care in Australia [9, 10], Finland [11], the Netherlands [8, 12], Austria [13], Denmark [14], Italy [15] and is also currently being translated into Japanese. In Australia, ASCOT is also being piloted as a potential quality indicator for aged care services [16]. Thus, its use as a tool for measuring the outcomes of long-term care and informing policy and practice is well established.

A number of different measures can be derived from the ASCOT toolkit [17], making it possible to estimate the impact a service is having on a person’s SCRQoL. The first, *current SCRQoL*, reflects the person’s currently experienced SCRQoL (with services in place). The second, *expected SCRQoL,* is an innovative method for estimating the counter-factual without the necessity for a control group [18] and reflects the SCRQoL that would be expected in the absence of services. Although this approach requires further validation and testing, early findings lend support for use of the counter-factual estimation approach in general [18] and more specifically, as a measure of expected SCRQoL in ASCOT [6]. Furthermore, in a previous care homes study, criterion validity of the expected scores was supported by the finding that nursing homes had significantly lower scores than residential care homes [19].

By subtracting *expected SCRQoL* from *current SCRQoL*, we can calculate the *SCRQoL gain,* which reflects the total benefit of the intervention or service [6].

$$ Current\kern0.3em SCRQoL\kern0.3em {\textstyle \hbox{-}}\kern0.3em expected\kern0.3em SCRQoL=SCRQoL\kern0.3em gain $$

Like most quality of life measures, ASCOT was originally designed as a self-completion tool but there are well-documented difficulties of using self-completion questionnaires and even structured-interviews with the most impaired populations [20–22], such as those living in long-term care. To overcome these issues, we developed a multi-method approach to evaluating the outcomes of care home residents [23, 24]. The care homes toolkit (CH3) collects data through structured observations and interviews, which then form the basis for ratings of residents’ SCRQoL [25]. In each domain, one rating is made to reflect whether people have no, some or high unmet needs in that aspect of their life. These are defined in Table 2. Some needs have a negative impact on the person’s quality of life, whereas high needs have consequences for the person’s physical or mental health. For example, in the case of food and drink, people who do not have meals at times they would like or choice over what to eat would have some needs; those who were getting an inadequate diet or insufficient liquids would have high needs.

**Table 2:** Outcome states for each ASCOT domain for current SCRQoL

Outcome state	Definition
No needs	The individual has no or the type of temporary or trivial needs that would be expected in this area of life of someone with no impairments.
Some needs	Some needs are distinguished from no needs by being sufficiently important or frequent to affect an individual’s qualify of life
High needs	High needs are distinguished from some needs by having mental or physical health implications if they are not met over a period of time. This may be because of severity or number.

CH3 was developed and tested in a study involving 366 residents living in 82 English care homes and showed acceptable properties [19]. Local fieldworkers were recruited and trained for the original study and inter-rater reliability showed acceptable percentage agreement (77 % for current and 81 % for expected SCRQoL) but kappa statistics suggested some room for improvement (0.47 for current and 0.57 for expected SCRQoL) [19]. Since its development, CH3 has been refined and the training and guidance improved, with a view to improving inter-rater reliability [26]. There has also been support from providers that ASCOT has the potential to have a positive impact on how services assess and meet residents’ health and social care needs [27, 28]. Unlike the self-completion and interview instruments, the observational element of CH3 provides context/evidence for the overall scores. Ratings are supported with real life examples taken from observations and interviews and there is scope to use these to help staff understand how they might improve the SCRQoL of residents [29].

However, we know from previous research that the process of improving practice is not straightforward [30, 31] and observations of practice can be perceived as threatening by staff [31, 32]. It is imperative the staff feel supported by management [33] and that any feedback delivered about ratings of residents’ SCRQoL and the context behind those ratings are delivered sensitively [31]. As well as the relevance and quality of the feedback intervention itself, there are several home-level factors that will influence its impact on practice. These include: the level of staff engagement with the research [34] and corresponding attendance at the feedback sessions [35, 36]; the care home culture [37]; and the skills and commitment of the management team [38]. Drawing on the key messages from research examining the impact of staff training on practice improvement [39], there is some evidence that training works best when it is tailored to the issues identified in particular settings, part of a wider commitment to quality improvement [33] and given to supervisory staff and management as well [40]. Although this intervention was considered feedback, not training, it was important to consider these key messages in this research. As such, following consultations with key stakeholders; including academics, a national care home provider and a representative from a UK-wide initiative to promote change and improve quality of life in care homes, this study worked with four care homes to design a feedback-intervention based on the measures included in the ASCOT. We examined whether the feedback was considered relevant and helpful by staff and also whether it led to any reported changes in practice and/or measurable improvements in the SCRQoL of residents.

### Aims and objectives

The aims of this study were to:

1. Design a feedback-intervention based on the evidence collected using the CH3 toolkit (observational notes and interviews) and pilot it in a small sample of care homes in England.
2. Examine the acceptability of this feedback to care home staff and explore whether there were any reported changes in staff practice and/or measurable changes in residents’ SCRQoL after the feedback had been delivered.
3. Examine and report new inter-rater reliability analysis on the CH3 approach

## Method

This study was funded by the School for Social Care Research and given a favourable ethical review from the Social Care Research Ethics Committee (12/IEC08/0051) who confirmed it complied with the requirements of the Mental Capacity Act [41].

### Homes and participants

As care homes and care home residents are known to be very difficult to recruit [24] with high attrition rates [42], we worked closely with four case study homes for older adults in one local authority in England. There are two main types of care home in the UK, residential care and nursing homes. All homes provide care and support throughout the day and night and have staff who provide help with washing, dressing, meal times and using the toilet. However, nursing homes also provide 24-h medical care from a qualified nurse [43]. In order to explore the feasibility of the intervention working in different types of homes, we purposively recruited two nursing homes and two residential homes. We also recruited both a large national chain and a small independent provider. Ideally each provider would have volunteered a home with and a home without nursing but our final sample included two nursing homes owned by a national care home provider and the two residential homes run by a small [44] independent provider. All homes accepted people living with dementia. Homes varied in size between 29 and 64 beds. The two residential care homes taking part in the study were unusual in that they only accepted female residents.

All staff were invited and encouraged to take part in the research. As the feedback was aimed at staff, we wanted as many staff as possible to be present. The feedback was relevant to all staff; including administrative, catering, domestic and estate. Family members were invited to take part in focus groups (to be reported elsewhere) and were asked their opinions of their relative’s SCRQoL, if they consented to be interviewed.

All permanent residents were invited to take part in the research, including people with dementia, other cognitive impairments and communication difficulties. The only exclusion criteria were those who were there for respite/short-term care and those currently in hospital. In accordance with the Mental Capacity Act [41], residents assessed as lacking the capacity to consent to take part in the research were recruited via the advice of a personal consultee. The Act defines a personal consultee as an unpaid carer or someone interested in the person’s welfare (such as a friend or relative), who is willing to be consulted [41]. We asked home managers for advice on this and where they felt consultees ought to be involved, they forwarded the appropriate information sheets and consent forms to consultees on our behalf. Alongside this, researchers spent time in each home talking to residents, explaining the study and assessing their capacity to consent. Throughout the study researchers continuously monitored whether or not residents agreed to participate. Consent was considered a continuous process and researchers continuously assessed residents’ willingness to be involved in the study (see [45]).

### Data collection

For the purposes of examining inter-rater reliability, two researchers collected the data for 84 % of the participating residents in the care homes at time one (T1) and again for 93 % of the residents at time two (T2). One was the main rater (R1) and provided the ratings on which all the analysis is based. The other was the second rater for reliability purposes (R2) and was also the main researcher preparing and administering the feedback. R1 did not prepare or administer the feedback, so whilst she was not blind to the T1 ratings, she was less likely to be influenced by what had been discussed during the feedback session when making the T2 ratings. R1 was trained by the lead author and R2, who are both ASCOT trainers. Checking reliability again at T2 allowed us to examine whether being part of the feedback biased R2 towards more positive ratings at T2 and whether R1, who was previously new to the ASCOT toolkit, agreed more or less with R2 after gaining experience during wave 1.

Following the same approach used in Netten, Trukeschitz et al. [19], staff provided information about residents’ functional abilities and their level of cognitive impairment through the completion of user characteristic questionnaires. At T1 and T2, researchers spent up to 5 days in each home using the CH3 toolkit. For each participant, the following data is collected using the mixed-methods toolkit: structured and general observations in communal areas, including during a meal time; conversations or interviews with the residents themselves (depending on their cognitive ability); and structured interviews with care staff asking them to respond to the ASCOT questions on behalf of the resident (proxy interviews). These data, together with detailed guidance, were drawn on in order to rate residents’ SCRQoL using the Adult Social Care Outcomes Toolkit (ASCOT).

To reflect the impact of the care provided by the home, CH3 includes both ‘current’ SCRQoL (i.e. experienced/achieved quality of life now) and ‘expected’ SCRQoL (i.e. expected quality of life in the absence of the care and support they receive in the home, holding all other factors constant). In care homes for older adults, where physical and cognitive decline is highly likely [46], we would expect to see a decline in residents’ *expected* SCRQoL if measured over sufficient time, as their ability to meet their own needs worsens. Thus, all other things being equal, if current SCRQoL remains constant, despite declines in expected, the resident is gaining more from the service over time; the service is increasingly compensating for their loss of functionality (ability to care for themselves). SCRQoL gain is a measure of the impact of care defined as the difference between current SCRQoL and expected SCRQoL.

### Measures

The main outcome measure is current SCRQoL, as measured by ASCOT. However, in order to understand these scores, we are also interested in trends in the expected SCRQoL scores and overall gain at T1 and T2. Rather than a simple summed score assuming each domain is of equal importance, the current and expected ASCOT ratings were weighted to reflect English population preferences [6]. Possible scores range from 1.0 to -0.23. The ASCOT score for each case is calculated by anchoring the score to the best possible or ‘ideal’ state and to the equivalent of ‘being dead’ state. This means that whilst a score of 1.00 would represent optimum or ‘ideal’ SCRQoL, a score of 0.00 would indicate a state that is equivalent, according to the preferences exhibited by the general population, to being dead. The score also can drop below zero into negative values. A negative score represents SCRQoL that is so bad that it is considered to be worse than being dead [6, 17, 47].

Inter-rater reliability was assessed using a two-way random, absolute agreement, single-measures Intraclass Correlation Coefficient (ICC) [48] to assess the degree that coders provided consistency in their ratings of SCRQoL across residents. The resulting ICC for current SCRQoL was good (ICC (2,2) = 0.72) [49] at T1 and excellent (ICC (2,2) = .76) at T2. Similarly, the resulting ICC for expected SCRQoL at T1 was good (ICC(2,2) = .71) and excellent at T2 (ICC(2,2) = .81). These ICCs indicate that coders had a good degree of agreement and that R1, who had not previously used the ASCOT toolkit, agreed more closely with R2 (an ASCOT trainer) after gaining experience of using the toolkit in the first wave.

**Fig. 1:**
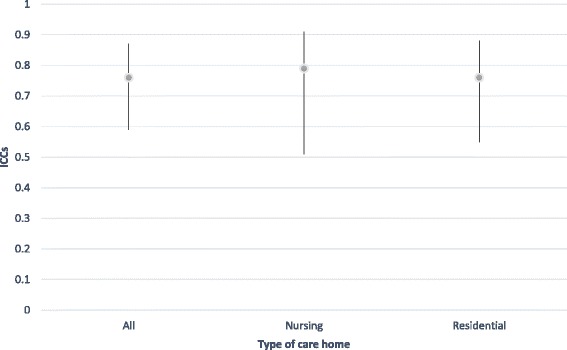
Comparison of inter-rater reliability for current SCRQoL at time 2 by type of home

**Fig. 2:**
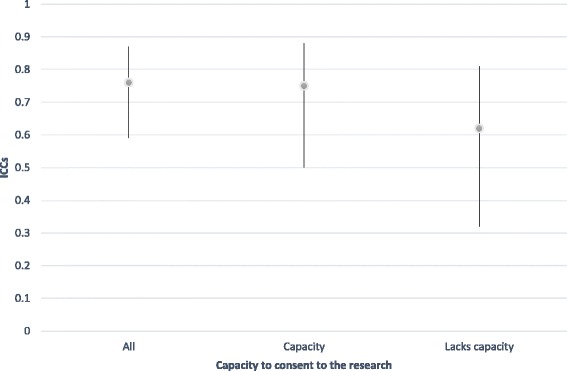
Comparison of inter-rater reliability for current SCRQoL at time 2 by the capacity of residents to consent

**Fig. 3:**
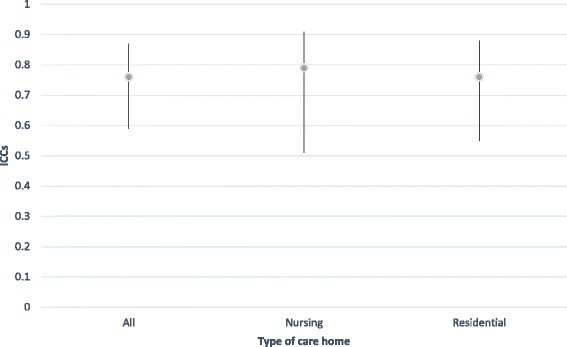
Comparison of inter-rater reliability for expected SCRQoL at time 2 by type of home

**Fig. 4:**
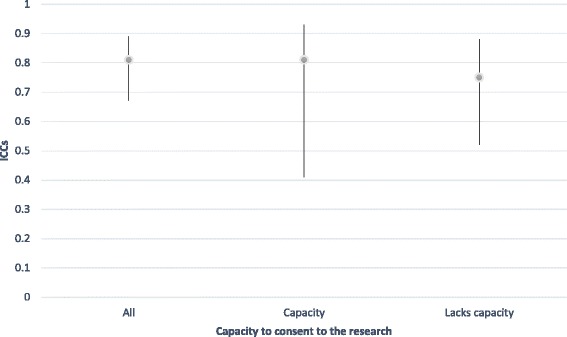
Comparison of inter-rater reliability for expected SCRQoL at time 2 by the capacity of residents to consent

We also examined inter-rater reliability for interesting subgroups within our sample using the time 2 data, in which we had a very high level of agreement overall. These are reported in Table 3. Notably, for current SCRQoL, there was very little difference in agreement according to type of home but less agreement for residents lacking the capacity to consent (IICC (2,2) = .62), although this is still considered a ‘good’ level of agreement [49]. For expected SCRQoL, we found the opposite pattern, with slightly less agreement in residential care home compared to nursing homes, however, it was very small and all ICCs were excellent (above .75) [49]. Following the approach outlined by Stolarova et al [50], differences between ICCs for different subgroups were evaluated by examining their confidence intervals (CIs), reported in Figs. 1, 2, 3 and 4. Overlapping CIs indicate that the ICCs do not differ significantly from each other.

**Table 3:** Comparing inter-rater reliability at time two for subgroups of interest

	ICCs Current SCRQoL at T2	ICCs Expected SCRQoL at T2
All homes	.76	.81
Nursing homes only	.79	.84
Residential homes only	.76	.77
Residents lacking the capacity to consent	.62	.75
Residents with the capacity to consent	.75	.81

These levels of ICC suggest that only a small amount of measurement error was introduced by the independent coders [51], and therefore statistical power for subsequent analyses is not substantially reduced. Both the current and the expected SCRQoL ratings were therefore deemed to be suitable for use in the analysis. There was no evidence of rater 2 bias at T2, despite him delivering the feedback in the homes.

### Feedback intervention

Feedback was based on the SCRQoL of participating residents. Scores for each domain were presented at an aggregate level to protect the anonymity of specific residents. Rather than focusing on the summed, preference-weighted scores, homes were given feedback at the domain level, supported by examples of how the researchers came to those ratings. The feedback sessions were held two weeks after T1 data collection. Feedback sessions began by describing how many residents took part in the research and reminding staff that part of the study was to explore whether the information was accurate and helpful. The session focused first on what the home was doing well. This would be the domains of quality of life where *most* residents had no needs and *no* residents had high needs. We backed these ratings up by giving examples from fieldwork observations. Afterwards, we discussed the domains where larger numbers of residents had some or high needs and gave examples to support these ratings too. See Table 4 for an example.

**Table 4:** Current SCRQoL ratings for ‘occupation’ in one case study home and an example of the feedback given to staff about this domain during the feedback sessions

Current occupation		
	Number of residents	% of residents
No needs	10	50
Some needs	8	40
High needs	2	10
*Feedback* • Just under half of the residents spent their time doing things they value and enjoy • Reading • Exercise sessions • Just under half did some of the things they enjoyed but not enough • Long periods with no activity but did something later or we were told about other activities they do • A few residents who had high needs – did almost nothing they enjoyed • Resident did no activities during observation and staff confirmed they do not do anything they value or enjoy. Another resident says she feels very bored and clearly states she does nothing.		

The researchers prompted group discussion by asking whether the feedback was representative of life in the home and how they felt about it. Throughout, staff were encouraged to think of ways to improve ratings and overcome difficulties in the domains of quality of life requiring improvement. To give all staff the opportunity to take part in the feedback we ran multiple feedback sessions throughout the day, as required by the homes. Feedback sessions were tape recorded and transcribed for analysis of the acceptability and face-validity of the feedback.

### Analysis

Data were analysed using a variety of non-parametric techniques appropriate for variables that do not have a normal distribution (e.g. ordinal variables). For comparisons between participants in homes with and without nursing, the Mann-Whitney U-test was used. When controlling for co-variates of type of home, such as dependency and cognitive ability, a General Linear Model was used instead. Chi-squared (X^2^) tests of association were used to explore relationships between capacity to consent and setting. For comparisons between T1 and T2, the Wilcoxon signed-rank test was used. To explore relationships between background variables and outcome variables, non-parametric tests of correlation were employed (Spearman’s rho). The statistical analyses were undertaken using SPSS Statistics, version 20 [52].

## Results

### Residents’ characteristics

Fifty eight residents across four homes were recruited to the research. Response rates ranged from 23 % in one of the nursing homes to 54 % in one of the residential care homes. This is consistent with previous research involving care homes for older adults in the UK [53]. Nobody withdrew from the study, however, we had to exclude nursing home 2 from the T2 data analysis of SCRQoL because the home was taken over by another provider and residents were being moved to other homes. Allowing for this, our attrition rate was 16 %.

The proportion of residents in our sample lacking capacity to consent was quite high (mean 53 %) but not surprising given that in excess of 80 % of care home residents in the UK have dementia or significant memory problems [54]. In one home, which was for older adults with nursing needs and dementia, the manager requested we involve personal consultees for all residents. Significantly more of the nursing home residents lacked the capacity to consent to the study (χ^2^(1,58) = 14.70, *p* < .001) compared with those living in homes without nursing. The impact of recruiting participants via consultees is explored in the discussion section. Nobody with capacity at T1 was found to have lost the capacity to consent at T2 (12 weeks later).

Of our total sample, 85 % were female, which is higher than that found in the population of older people living in care homes [55]. As shown in Table 5 and outlined above, this was because two of the homes in our sample were exclusively for female residents. Residents ranged in age from 73 to 97 years old with a mean age of 86 years. Age did not significantly vary by type of home (*p* = 0.34). 60 % of our sample were self-funding their own care, 10 % were part publicly funded and 19 % were completely publicly funded. We had missing information for the remaining 11 %. All our sample were white and 97 % were White British/Irish, which is in line with 2011 census data on the over 65 s in the South East of England, reporting over 97 % of the population in this area as being white [56].

**Table 5:** Characteristics of homes and residents

Variable	Home 1	Home 2	Home 3	Home 4	All
Home characteristics					
Type	Nursing	Nursing	Residential	Residential	-
Size	64	39	37	29	-
Provider	National chain	National chain	Small independent	Small independent	-
Resident characteristics T1					
*N*	15	9	20	14	58
*N* female (%)	9 (60 %)	6 (67 %)	20 (100 %)	14 (100 %)	85
% White British/Irish	87	100	100	100	97
Age range (min-max)	77–96	73–93	74–97	73–94	73–97
Mean Age	87	82	87	86	86
% lacking capacity	100	44	30	36	53
Mean Bathel Index Daily Living	8.00	4.44	10.55	9.43	8.67
Mean MDS CPS (0-6)	3.80	3.67	3.00	3.36	3.40
Resident characteristics T2					
*N*	15	7^a^	20	14	56
% lacking capacity	100	43	30	36	52
Bathel Index Daily Living	6.20	6.00	8.80	9.29	7.88
MDS CPS	3.80	3.86	3.05	3.50	3.46

^a^Although SCRQoL data is not reported for T2 in home 2, we do have data from the home about the characteristics of 7 residents during that time and these are reported here

The Barthel Index of Activities of Daily Living [57] was calculated (see Table 5). Scores range from 0 to 20, with higher scores indicating greater independence and lower scores indicating the need for more help. Nursing home residents had significantly (U = 271.50, *p* < .05) lower T1 scores and T2 scores (U = 265, *p* < .05) than those in homes without nursing. The overall sample mean was 8.67, which is lower than in previous research indicating greater levels of dependency [58, 59]. The mean Barthel score for the whole sample declined significantly between T1 and T2 (z = -2.67, *p* < .01).

Cognitive impairment was measured by the Minimum Data Set Cognitive Performance Scale (MDS PS) [60], with scores ranging from zero (intact) to 6 (very severe impairment). The mean score for the whole sample was 3.4 at T1, which is higher than previous research [58, 59] and may reflect the increasing incidence of cognitive impairment in care home residents and/or the ability of our methodology to include them in the sample (Baumker et al, 2011 and Darton et al, 2012, excluded residents lacking capacity to consent). There was no change in mean cognitive impairment score between T1 and T2 (z = -1.63, *p* = .10). Nursing home residents were significantly more cognitively impaired (median = 4.00) than residents in homes without nursing (median = 3.00) (U = 276.00, *p* < .05).

### Social care-related quality of life

Mean scores for current SCRQoL in our sample were 0.71 at T1 and 0.67 at T2 (See Table 6). This difference is not significant (Z = -1.49, *p* = .14). Decline in residents’ mean expected SCRQoL between T1 (.13) and T2 (.06) was significant (z = -2.41, *p* < .05), indicating that, at T2, they were less able to meet their own needs without help from services. This is in line with the reported decline in residents’ abilities to perform activities of daily living and in fact the two scores are significantly correlated at both T1 (rs = .69, *p* < .001) and T2 (rs = .58, *p* < .001). However, despite residents’ requiring more help at T2 to maintain similar levels of SCRQoL, the overall gain from services remained the same (z = -.29, *p* = .77). This is due to the fact that current SCRQoL also dropped slightly, albeit not significantly. Neither current SCRQoL (rs = .13, *p* > .05, NS) nor expected SCRQoL (rs = -.11, *p* > .05, NS) was even marginally related to residents’ age in our sample. We were unable to reliably test for gender differences, given the very small number of men in our sample (*N* = 10).

**Table 6:** Showing SCRQOL scores (current, expected and gain) for the homes in our sample

	TI Current	T2 Current	T1 Expected	T2 Expected	T1 Gain^a^	T2 Gain
All homes						
*N*	58	49^a^	58	49^a^	58	49^a^
Mean	.71	.66	.17	.06	.57	.60
SD	.21	.23	.25	.15	.23	.17
Min	.06	.06	-.11	-.11	.01	.11
Max	1	1	.88	.45	1.05	1.05
Home 1 (dementia nursing)						
*N*	15	15	15	15	15	15
Mean	.62	.54	.10	.03	.52	.51
SD	.27	.25	.22	.14	.24	.19
Min	.06	.06	-.11	-.11	.11	.11
Max	1	1	.41	.26	1.05	.82
Home 2 (nursing)						
*N*	9	0	9	0	9	0
Mean	.54	NA	.05	NA	.50	NA
SD	.19	NA	.12	NA	.28	NA
Min	.27	NA	-.07	NA	.07	NA
Max	.82	NA	.29	NA	.84	NA
Home 3 (residential)						
*N*	20	20	20	20	20	20
Mean	.77	.74	.20	.11	.57	.63
SD	.17	.17	.29	.17	.25	.11
Min	.42	.33	-.11	-.11	.01	.44
Max	1	.93	.88	.45	.91	.77
Home 4 (residential)						
*N*	14	14	14	14	14	14
Mean	.72	.68	.05	.01	.66	.67
SD	.20	.24	.20	.12	.14	.19
Min	.44	.36	-.11	-.11	.42	.40
Max	1	1	.46	.22	1.05	1.05

^a^Home 2 was not included in the T2 data because during T2 data-collection the home was being taken over by the NHS and residents were in the process of moving

Using the T1 data for all four homes, residents living in homes without nursing had significantly higher SCRQoL scores (median = .74) than those living in homes with nursing (median = .65) (U = 258.50, *p* < .05). However, after controlling for the differences in residents’ needs and characteristics related to setting (Barthel and MDSCPS), this difference in SCRQoL no longer held (F(2,58) = 3.60, *p* = .06).

### Acceptability of the feedback intervention and reported changes to practice

The acceptability of the feedback intervention was explored in both the feedback sessions with staff and in interviews with home managers after T2 data collection. What emerged was generally a positive view from staff and managers on the data collection process and feedback intervention:

*“There was no disruption to the home at all, [The fieldworkers] just went off, found their residents that they needed to observe, and just basically just took hold of it all and got on with it. It didn’t cause any disruption to us whatsoever.” (Manager Nursing Home National Chain)*

The interviews with staff about residents’ SCRQoL, were deemed a strain on staff time, although staff were not uncomfortable with researchers being present to observe:

*“The staff were actually fine because the staff are used to people coming in and out…. everybody seemed to be very discreet. I mean, you know, so if they were aware they forgot that you were there.” (Manager Care Home Independent)*

Any apprehension staff may have felt at the start of the research disappeared as staff realised that fieldworkers were not there to scrutinise or criticise them and their working practices. During the feedback sessions, staff often expressed support for our findings and in one case a desire that the research team ensure that management were made aware of our findings. Some staff were also happy to think about what the findings meant for residents and how they could address the issues our work had raised:

*Interviewer: “I just wondered how useful you found this feedback?…”*

*Staff 4: “I think it is actually ‘cause we.. where we are, so I’m like constantly from one job to the next job,… sometimes it takes outside eyes … to see that” (feedback session Nursing home national chain)*

Staff and managers agreed with the feedback they were given and felt it accurately reflected the areas of quality of life they do well at (personal cleanliness and comfort, accommodation cleanliness and comfort, safety and dignity) but also identified areas they struggle to make time for (choice over food, control over daily life, social participation and occupation):

*Staff 1: “Well everything you said about the activities is completely right, it’s not enough” (feedback session nursing home, national chain).*

All sessions led to interesting conversations about care workers’ desires to meet these needs but the challenges they faced within their organisational culture to do so. In the nursing homes in particular, which were both owned by a large national provider, staff frequently talked about insufficient staff-resident ratios. They felt they could meet the basic health and social care needs but did not have time for anything else:

*Staff 4: “Unfortunately, you know, unless or until [provider] ups their [staffing] levels where--, ‘cause at the minute--, I mean as it stands at the moment we are five residents per one member of staff”*

More quotes representing the issues that arose are summarised in Additional file 1: Table S7, which can be found in the Additional file, with reference to the domains being discussed in each case.

Staff from the nursing home for people with advanced dementia were particularly positive about the focus on quality of life because they felt their emphasis was usually on meeting health/nursing needs and that the organisation they worked for did not employ enough staff to do more than get people up, washed, dressed and fed:

*Staff 2: “They always say, don’t they, when you go to a care home you always see residents in the lounge asleep? It’s not because they’re tired, it’s ‘cause they’re bored, it’s boredom I think a lot of the time.”*

*Staff 1: “There’s nothing keeping their mind going.”*

*Staff 2: “Exactly, what do you do? You sleep when you’re bored.” (feedback session, nursing home, national chain)*

There was a sense that the feedback gave them an opportunity to raise issues around staffing structures and the limitations this placed on their ability to spend time with residents, socialising and supporting their independence:

*Staff 2: “Will it go past regional management?”*

*Interviewer: “I don’t know.”*

*Staff 2: “I hope so, I hope so.”*

Indeed, it was in this home that the feedback had the biggest impact of reported changes to practice:

*“I completely changed the whole setup of the working day. So I looked at smaller groups of residents, because the staff were coming back to me and saying, ’We haven’t got time to complete all of our tasks with so many residents.’.... They now have more time to spend with the residents in terms of social care; the little things, painting nails, and so on and so forth, and the lipstick and it’s all very, very important. So that took the onus off of a task-orientated workload.” (Care Home Manager Nursing National Chain)*

Interestingly, despite these changes, the manager was not certain it had impacted upon the residents:

*Interviewer: “Have you noticed any changes in residents from those changes?*

*Manager: “It’s difficult to say with the residents. I mean there are a few that are happier now that they have got their time set for them in the morning.” (Care Home Manager Nursing National Chain)*

This perhaps reflects the challenges of maintaining measurable or observable improvements in quality of life in long-term care settings, particularly nursing homes where many people have multiple comorbidities and complex needs.

Nevertheless, all managers felt that they had been able to use our feedback to put in place changes in the home that they hoped would improve quality of life for the residents. For example, feedback about low levels of occupation/engagement led to the residential care home provider employing an organisation specialising in creating activities for older adults with dementia:

*“they’re doing some training here. It is interesting. It’s broken up into different--, a whole series of different modules from kind of meet and greet, icebreaker type things to physical activities, to singing, to storytelling, erm, and it’s all themed and it kind of allows the--, it allows the residents to sort of take things off in a particular direction.” (manager, residential care, independent provider)*

Managers also reflected upon the acceptability of the whole process of the feedback intervention to residents and their families and friends. Again the view was broadly positive, with no notable differences between the nursing homes and the residential care homes. One manager suggested that subtle and discreet observational techniques by fieldworkers meant that we did not affect either residents’ “day to day routine” or “their relationships with anybody else that’s in the environment” (Residential care Home manager independent). However, it was noted by the manager of one of the nursing homes that some residents on the more severe dementia floor did display “some extra agitation” whilst we were there. This was not something noted by the other three homes (including the other nursing home) or by the researchers themselves during the visits. However, there was a higher degree of cognitive impairment and disability in this particular home because the research was carried out on the floor for people with advanced dementia and nursing needs. It will be important to explore the impact of researchers being present in such settings in future research.

In terms of acceptability to relatives and friends of residents, managers felt that they were overwhelmingly supportive of the research project:

*Interviewer: “How about relatives and residents? Did they have any comments to make about it [the research]?”*

*Manager: “… from the very onset, once they had their letters explaining to them what was going to happen, they were quite enthralled by it and they were looking forward to actually having an outside person come and look at what it is that we do here at [the nursing home]. So they were on our side from start to end.” (Nursing Home Manager National Chain)*

Low but not unusual recruitment levels meant that there were concerns about those who did not take part in the research directly. One manager addressed this in the interview saying that some of the relatives of residents who lacked capacity did not want their relative to take part directly in the research due to concerns that it might cause them stress. Nonetheless, the manager did feel that there was still general support for the project from these relatives despite not wanting direct participation by their relative who lived in the home:

“*I mean although there was a tiny minority of relatives that didn’t want their relatives being in the research there was no concern about you being there and they actually felt the research itself was of value… they just didn’t want their own--, they didn’t want you questioning or asking their relatives ‘cause they thought it might cause them distress, but there was no concern about you being there to do it “ (Care Home Manager Independent)*

## Discussion

This study sought to design a feedback intervention based on the observational and interview evidence collected by the mixed-methods approach to measuring outcomes in care homes using the Adult Social Care Outcomes Toolkit (ASCOT). We worked in partnership with care home providers to design an intervention and agreed its implementation in four case-study homes. The pilot study aimed to collect revised estimations of inter-rater reliability for the CH3 toolkit and explore the acceptability and feasibility of the intervention to care home staff.

Recruitment of residents was a significant challenge throughout this research, which worked with two nursing and two residential homes, all of whom cared for at least some people living with dementia. For example, in one nursing home for people with dementia, all residents lacked the capacity to consent and had to be recruited to the study via the advice of personal consultees. This places considerable administrative burden on the homes, who, for data protection reasons, have to act as gatekeepers to those consultees, sending information sheets to family members and representatives on our behalf. This effectively means that the pathway between the researcher and the resident is at least four steps (researcher-home-consultee-researcher-resident), making the recruitment process very long and slow. We also experienced difficulties with one of the nursing homes, who unexpectedly transferred its ownership to the NHS after the feedback stage of the project and had to withdraw from the research. This home also experienced a complete change of senior management during the life-cycle of the project, which affected the support the research project received in contacting consultees and recruiting residents in that home.

Nevertheless, once we had consent to collect data in the homes, we found staff and residents welcoming and cooperative. In line with previous research [19], resident outcomes were higher for the basic quality of life domains and most of the feedback sessions focussed on the higher order domains: control over daily life, social participation and occupation, with one home also having some needs around choice of food and drink. Staff responded well to the feedback in all homes and felt it accurately reflected what they did well, as well as the areas they found more challenging to address. In the nursing homes in particular, staff felt that they were restricted by low staff numbers and a task-focused approach to caring. In all homes, staff engaged well with the feedback sessions and came up with ideas to improve outcomes for residents.

However, despite staff finding the feedback helpful and valid, and managers saying they had implemented changes because of it we did not find a measurable improvement in SCRQoL between T1 and T2. It seems likely that our follow-up time of 12 weeks was not long enough to elicit improvements in residents’ SCRQoL. This period was agreed following consultations with senior management within the homes, who felt this should be enough time for changes in practice. However, previous work on the impact of interventions in residential care has noted that three months might not be long enough to see improvements in such things as unmet need, quality of life or depression [61, 62]. Moreover, the procedures of using the well-established Dementia Care Mapping Tool (DCM) [63, 64] in studies of care practice tend to suggest gaps of one year between periods of data collection [65, 66]. Indeed, in one of the homes in this study, a new approach to engaging residents in activities, being trialled in response to our feedback, had only just begun on the last day of T2 data collection. It had, therefore, had no opportunity to impact upon the lives of residents but may have done had we been able to go back again at a later date.

Whilst this may explain why we might not see an improvement in current SCRQoL between T1 and T2, it does not account for the slight decline in residents’ current SCRQoL. To understand this, it helps to look at the *expected SCRQoL* scores. Across all homes, expected SCRQoL decreased between T1 and T2, matched by a significant decline in Barthel scores, indicating that those taking part in the study became increasingly frail between T1 and T2. Despite significant declines in health and expected SCRQoL, current SCRQoL declined only slightly because homes have compensated (or at least partially compensated) for this by adapting/increasing the care and support. This has implications for the measurement of SCRQoL and how we judge social care interventions. Most people using social care services have conditions that involve a permanent (and often declining) loss of functional ability. In these situations, the primary aim of social care interventions is to compensate a person for their lost functional ability, rather than try to restore it [1]. Whilst we expect good services to meet residents’ needs despite these challenges, in care homes the decline is often rapid [46] leading to frequent fluctuations in health and social care related quality of life. During this study, researchers often rescheduled interviews and observations with individual residents because of poor health and noted that residents have ‘good and bad days’. If observing on a bad day, ratings might indicate a lower than average outcome for that individual. If observing on a good day, the opposite might be true. Methodologically, this is a limitation of measures relying on ‘snapshots’ of information about residents’ lives.

One alternative approach would be to integrate outcome measurement into care planning, so that variation in health and social care needs can be accounted for and outcomes improved in a targeted, person-centred way. Arguably, had staff collected the data and made their own ratings of residents’ lives, using ASCOT, it may have had more impact on care practice than a feedback intervention and would also have had sustainability beyond the life of the study, providing potential for ongoing benefits for residents and staff. Although subject to several drawbacks from a research perspective, not least the loss of an external independent evaluation of residents’ lives on which to base the ratings and feedback, the model is attractive to care providers. Since conducting this research, one national health and social care provider in England has integrated ASCOT into their care planning processes with a view to improving the quality of life of service users [28] and another provider in New South Wales is also piloting this approach. Future research will aim to examine the reliability of staff ratings of residents’ SCRQoL compared with judgements made by external researchers/fieldworkers. In this study inter-rater reliability was excellent but nonetheless showed an improvement over time, emphasising the value of practice and experience in making these ratings, particularly for people who lack the capacity to consent. Furthermore, comparing SCRQoL in homes adopting this outcomes-focused approach to care planning with matched homes following usual care planning will help us evaluate the impact of this approach in residents’ quality of life.

## Conclusion

The older participants in our study declined significantly in terms of their health and social care needs during the three-month period between giving the feedback and collecting the follow-up data. Despite this, their overall SCRQoL remained largely the same. Thus, homes maintained residents’ quality of life but did not improve it. As this was a small feasibility study, it did not include a control group, and so we cannot draw any conclusions about whether the feedback had a role to play in this. Furthermore, a limitation of our results is that they are based on a very small sample, reflecting the difficulties we had recruiting and retaining homes to the research. However, the ASCOT feedback was well-received, considered valid by staff, and changes in practice were reported by managers. Furthermore, since conducting this research, one national and one international provider have begun integrating ASCOT into their care planning and review activities, indicating the growing support for this approach within the sector and its relevance to an international audience. We have identified a variety of different options to address the problems raised and aim to address these as part of future research with these providers to evaluate the use of ASCOT in care planning.

## Additional file

Additional file 1: Table S7. Summary of staff responses and any reported changes in practice. (DOCX 15 kb)

## Abbreviations

ASCOT: Adult Social Care Outcomes Toolkit
SCRQoL: Social care-related quality of life
