# Supplementary material for: The acceptability and feasibility of using the Adult Social Care Outcomes Toolkit (ASCOT) to inform practice in care homes
Source: BMC Health Serv Res. 2016 Sep 29;16:523. doi: 10.1186/s12913-016-1763-1 (PMC5041329; doi:10.1186/s12913-016-1763-1)
Supplement: Additional file 1: Table S7. — Summary of staff responses and any reported changes in practice. (DOCX 15 kb) [file 12913_2016_1763_MOESM1_ESM.docx]

### Table 7: Summary of staff responses and any reported changes in practice

| **Examples of staff responses** | **Reported changes in practice** |
| --- | --- |
| **Home 1: nursing dementia (feedback focused on occupation, control, social and food and drink)** | |
| “I think from the feedback that you’ve given, I think [it’s] more or less spot on.”  “The thing is though, there’s 30 of our residents and there’s six of us to look after them.”  “[residents] are bored because we don’t have the time to devote to them.” (occupation)  “[some people] get put in front of the telly and that’s it, and it’s not enough” (occupation)  “we’ve got this urn and there are plenty of people that are very capable of making a cup of tea……even if one of us was just in there just to supervise” (control, food and drink, occupation)  “…and this is near enough on a daily basis, I say “Do you want a cup of tea?” and they say “are you going to have one with me then?” (social)  “I do think they should have more choice in the menus, I think the chef should just go down and speak to them as a group, you know, and say, “What foods do you really, really like?” You know, “Is there anything you really particularly fancy?” Or you know, I mean how hard is that, communication? How hard is that? It’s not hard.” (food and drink, control) | Re-organised the working day  Staff responsible for less residents  Dedicated staff for individual residents  Focus on people not tasks |
| **Home 2: nursing (feedback focused on control, social and occupation)** | |
| “at the end of the day, perhaps they want to feel like they want to go down the town and have a look round the shops and that’s--, and unless there’s plenty of staff in to help them do that and it’s agreeable they can’t do it, can they? [Pause] I don’t know.“ (Staff6, control)  “we were saying about activities, it’s quite hard in the morning to do other than what we do do, which is get them all out of bed and fed” (Staff1, occupation)  “they all--, some of them looked bored but they’ve--, we’ve--, you have to keep moving on, don’t you?” (Staff5, occupation)  Staff 4: “it is hard, isn’t it, sometimes ‘cause you think, yeah, I’d love nothing better than to go and sit for ten minutes--,  Staff 1: And have a chat--.  Staff 4: Do a bit of knitting.  Staff 1: Yeah, with them.  Staff4: Have a cup of tea, have a chat.  Staff 1: Yeah, it is difficult.  Staff 4: Unfortunately, you know, unless or until [provider] ups their [staffing] levels where--, ‘cause at the minute--, I mean as it stands at the moment we are five residents per one member of staff, so yeah…  “But from the social side of it I mean it’s--, you know, yes, we try and make sure if they’re in the room or in the lounge, you know, have they’ve got the telly on appropriately, the music’s appropriately in the bedrooms, but it’s the one to one time I think that—“ (Staff 4, social)  They’re doing bingo now but most of the residents are not really into it.” (Staff 8, social, control, occupation) | This home transferred ownership to the NHS after the feedback was given and all residents were moved, so there was no opportunity to measure impact. |
| **Home 3: residential (feedback focused on social, occupation, control) and home 4: residential (feedback focused on lack of personalisation in some bedrooms (accommodation), social, occupation and control)** | |
| “We are striving to improve all those all the time as well, find different methods to achieve that…cause the socialisation and the kind of engaging and that’s always much harder isn’t it?” (Staff 1, all domains)  Are there things that you can suggest, you know, from your, you know, that we could do that might, you know, practices that you know that can work and--, (Manager, occupation)  “the table needs laying, let’s get two or three people to help, that sort of thing” (Staff 2, occupation)  “Yes, there are some individuals that will talk if they are placed together, they don’t know that but you know that, you know, and that’s quite important to put those individuals together and then you know that they will actually initiate a conversation with each other, separate them out they don’t talk, you know, together they do.” (Staff 4, social)  “And there are some families unfortunately that don’t want, you know. They don’t want to come because they say, “Well they don’t recognise me so what’s the point?” “(Staff 3, social)  "there are things that don’t cost money, you know, being kind, being caring, talking to people, facilitating…creating friendship groups, yes…music and things like that.” (Staff 4, occupation, social)  I remember seeing one lady in once and she said, “I’m not bringing anything in with me because that’s it, my old life has gone, my new life is starting and I don’t want reminding of everything I’ve lost.” (Manager, personalising rooms)  “(talking about residents’ art) we usually put them out in the dining room which is where everyone’s stuff is and there’s also pictures of various ladies there which that’s going to be updated. But that is where sort of their lives now are usually focused. I suppose they could be moved on into their rooms once they’ve had their little showing in the dining room, that might be something to help (Staff 3, personalise rooms now) | Homes 3 and 4 were owned by the same provider and had similar feedback. The following changes in practice were reported for both homes:  Theatre company working with residents to improve activities/engagement and train staff.  Trying to implement culture change: becoming less task focused and giving residents time to communicate their wants and needs.  Supporting staff to listen, giving them permission to chat with residents.  Moving residents’ own artwork from the display in the dining room, into their own bedrooms when they update the displays downstairs. |
